# Caregivers’ Responses to Children’s Negative Emotions: Associations with Preschoolers’ Executive Functioning

**DOI:** 10.3390/children9071075

**Published:** 2022-07-19

**Authors:** Carla Fernandes, Ana F. Santos, Marilia Fernandes, Manuela Veríssimo, António J. Santos

**Affiliations:** William James Center Research, ISPA-Instituto Universitário, 1149-041 Lisbon, Portugal; csfernandes@ispa.pt (C.F.); afsantos@ispa.pt (A.F.S.); mfernandes@ispa.pt (M.F.); mveriss@ispa.pt (M.V.)

**Keywords:** emotion socialization, emotion regulation, executive functioning, preschool

## Abstract

There is a lack of knowledge regarding the connection between parental emotional responsiveness and children’s executive functioning (EF). This study aimed to explore the relations between caregivers’ reactions to their children’s distress and children’s EF. Mothers of 136 preschoolers reported their reactions to their children’s negative emotions using the Coping with Children’s Negative Emotions Scale. Children’s EF was assessed through the mothers and teachers’ reports using the Behavioral Inventory of Executive Functioning for Preschool Children. Results showed that the mothers’ perceived use of negative emotional regulation responses (i.e., punitive and minimizing reactions) was associated with lower levels of EF in children, as reported by both mothers and teachers. The association between the mothers’ use of positive emotional regulation responses (i.e., problem-focused, emotion-focused, and expressive encouragement reactions) and children’s EF was not significant. Multiple regression analyses revealed that the mothers’ use of negative emotional regulation responses accounted for significant proportions of variance in EF indexes. These findings suggest that parental socialization of emotion could be important for children’s EF. Specifically, caregivers’ negative emotional regulation responses to children’s distress may serve as a risk factor for poorer EF in children. Efforts to improve children’s EF may be more effective when parental emotional responsiveness to their distress is considered.

## 1. Introduction

The preschool years are critical for the development of executive functioning (EF) and emotion regulation, with important and rapid changes occurring during this period [1,2,3,4,5]. Even though a consensual definition is still lacking [6], EF generally is characterized as the set of cognitive higher-order processes involved in the self-regulation of thought and behavior and comprises the coordination of several capabilities [7,8]. EF involves three distinct but related functions: working memory, inhibitory control, and cognitive or attentional flexibility [8,9,10,11,12]. Working memory refers to the ability to retain and manipulate information in the memory [13]. Inhibitory control is the ability to suppress an automatic or dominant response, replacing it with a more adaptive one [14]. Lastly, cognitive or attentional flexibility reflects the ability to focus and shift attention when changes in stimuli or goals occur [15,16,17]. This function requires both working memory and inhibitory control, so it is considered one of the most advanced components of EF and thus develops later [8]. Although several other functions could be considered part of EF, these three are the most studied and agreed upon [6]. Additionally, as supported by Diamond [9], they also are the basis of other functions, such as reasoning, planning, or problem-solving, which are considered more complex executive functions. Like EF, there are numerous definitions of emotion regulation, but it can be broadly conceptualized as the interplay between extrinsic (e.g., parental emotion socialization) and intrinsic processes (e.g., child temperament) required to monitor, evaluate, and modify emotional experiences and expressions [18,19,20,21].

### 1.1. The Relation between EF and Emotion Regulation

EF and emotion regulation are both considered domains of self-regulation [22,23,24], and growing research has focused on links between them (e.g., [25,26,27,28,29,30,31,32]). Most studies emphasize the supporting role of executive functions in emotion regulation [33,34,35,36]. In fact, executive functions are considered top-down cognitive processes that support cognitive, emotional, and behavioral regulation, whereas emotion regulation is considered a bottom-up process that involves the experience, expression, and management of emotional experiences (e.g., [9,12,37,38]). In this sense, research shows that impairments in EF negatively impact emotion regulation [39,40,41,42]. However, several researchers suggest that cognition and emotion are interwoven, and bidirectional influences are likely [38,39,43,44,45]. This approach is supported by neurological research that suggests EF and emotion regulation may share neural mechanisms in the brain [39,43,46,47,48]. Particularly, the two major subdivisions within the anterior cingulate cortex (ACC) of the prefrontal cortex (PFC), which are responsible for cognitive and emotional processes, have been identified as reciprocal [26,43,44,49]. These relations suggest that EF and emotion regulation are intricately connected and influence each other. Thus, as supported by Carlson and Wang [39], emotions can contribute to the organization of thinking, learning, and behavior, and in turn, cognition contributes to the regulation of emotions.

Furthermore, it is well known that children learn to regulate their emotional experiences through interactions in their immediate environment, so caregivers play a crucial role in socializing children’s emotion regulation [21,50,51]. Similarly, research also shows that caregivers impact the development of children’s EF, namely through the interactions they establish with children (e.g., [52,53,54]). Given emotion regulation and EF are linked and that caregivers have a significant influence on children’s development of emotion regulation, it is also possible that parental socialization of emotion could be related to children’s EF [55].

### 1.2. Parental Reactions to Children’s Negative Emotions

According to Eisenberg, Cumberland, and Spinrad [56], one of the important components of parental socialization of emotion is caregivers’ reactions to children’s emotions. Emphasis has been placed on how caregivers react to children’s negative emotional expression [57,58]. Caregivers vary in their reactions, with research showing that they generally respond to their children’s negative emotions in a supportive or unsupportive manner [59]. Supportive responses, such as problem-focused responses, emotion-focused responses, and expressive encouragement, encourage children to express and discuss their emotions appropriately [56,60]. These types of responses are associated with early and later childhood socio-emotional competence, better emotion regulation skills, and lower levels of internalizing and externalizing problems [1,61,62,63,64,65,66,67]. On the contrary, unsupportive responses, such as distress responses, punitive responses, and minimization responses, communicate that emotional displays are undesirable or unacceptable and discourage children from expressing and discussing their negative emotions, which may result in difficulties managing their emotions appropriately [56,68]. Unsupportive responses are then associated with emotion dysregulation, lower socio-emotional competence, and the development of the internalizing and externalizing of problems [66,67,69,70,71,72,73,74].

As parental responses to children’s emotional expression are strongly related to children’s emotion regulation skills, and in turn, emotion regulation influences EF [44], we believe that it is also important to consider the contribution of these responses to children’s EF. Moreover, caregivers’ reactions to children’s negative emotions reflect caregivers’ emotional responsiveness, and responsive parenting is associated with advanced executive functions in children [53,54].

### 1.3. Current Study

To our knowledge, this is the first study to assess the relation between caregivers’ reactions to children’s negative emotions, using the CCNES [75], and children’s EF, using the BRIEF-P [76]. Only two studies have included both the CCNES and BRIEF-P. However, the only sub-scale used from BRIEF-P in these studies was the Emotional Control sub-scale, which was used to assess children’s emotion regulation, not EF functioning [55,77]. Even studies using different measures of EF tend to focus only on one single score or dimension, for instance, inhibitory control [78]. However, given its complexity, it is widely accepted that EF is better captured considering its multidimensional nature [79,80]. Thus, in this study, all sub-scales from BRIEF-P were considered. More specifically, we explored associations between the mothers’ reported responses to children’s negative emotions and the children’s components of EF as reported by mothers and teachers. The reports from teachers were also included because they could be more reliable reporters of children’s EF as the demand for children’s executive functions is higher at school than at home [81]. We hypothesized that mothers’ supportive emotion regulation responses to child’s distress would be associated with better EF in children, whereas unsupportive emotion regulation responses would be associated with poor EF in children. Additionally, as children’s executive functioning and the mothers’ responses to children’s negative emotions can be affected by different variables, namely demographic variables (e.g., [74,82,83,84,85]), we also examined possible differences regarding these variables.

## 2. Materials and Methods

### 2.1. Participants

Participants were mothers of 136 children. Children ranged in age from 2 to 6 years old (*M* = 3.60; *SD* = 1.14); 45.6% were girls and 54.4% were boys; 45.6% were firstborns, and 58.1% had siblings. Children were recruited from 6 schools from Lisbon and Cascais, in Portugal. They spend between 5 to 10 h at school (*M* = 7.97, *SD* = 1.11). Most of the parents (84.5%) were either married (46.3%) or cohabitating (38.2%), 4.4% were separated, and 5.9% in other situations. Mothers’ age ranged between 22 and 49 years (*M* = 35.46; *SD* = 5.53), whereas fathers’ age ranged between 24 and 59 years (*M* = 37.13; *SD* = 6.30). Regarding education levels, mothers’ ranged between 9 to 21 (*M* = 16.04; *SD* = 2.95), whereas fathers’ ranged between 5 and 21 (*M* = 14.76; *SD* = 3.44). Most of the parents work full time (mothers 81.6% and fathers 86.8%), 2.2% of both mothers and fathers work part-time, and 11.8 % of the mothers and 2.9% of the fathers were unemployed. For 87 of the children (46% were girls and 54% boys; age *M* = 45.49, *SD* = 13.12) teachers’ reports on their executive functions were available. The same teacher provided data for a maximum of 11 children (*M* = 5.73; *SD* = 2.71).

### 2.2. Measures

#### 2.2.1. Caregivers’ Reactions to Children’s Negative Emotions

Mothers’ responses to children’s negative emotions were assessed using the Coping with Children’s Negative Emotions Scale (CCNES) [75]. CCNES is a self-report questionnaire that measures parental (in our case mothers’) emotion socialization through 12 scenarios. All scenarios reflect typical situations in which children (from preschool to elementary school) experience distress and negative affect involving normative expressions of negative emotion for preschool (e.g., fear, anger, sadness, embarrassment, and disappointment). For each scenario, there are 6 possible parent responses to the children’s negative emotions. Parents are asked to answer each of six different ways using a 7-point Likert-type scale ranging from 1 = “very unlikely” to 7 = “very likely”. Four items are inverted. Three types of response are more related to supportive reactions, which imply comforting, encouraging emotion expression, and looking for solutions to problem (*Expressive Encouragement, Emotion-Focused Reactions, Problem-Focused Reactions*), and three others are more related to unsupportive reactions, implying distress manifestations, minimization of emotion importance, and punishing the child’s emotional behavior (*Distress Reactions, Punitive Reactions, Minimization Reactions*). Subscales were computed by averaging their respective items. *Distress Reactions* (DR) focus on the degree to which parents experience negative emotional arousal concerning their child’s negative emotions. *Punitive Reactions* (PR) reflect parents’ verbal or physical punishment-based and controlling responses to the child’s emotional expression. *Emotion-Focused Reactions* (EFR) addresses parents’ ability to help children feel better in an emotional situation. *Expressive Encouragement* (EE) refers to parental support for the child’s emotional expression. *Problem-Focused Reactions* (PFR) reflect parents’ scaffolding and supporting children in problem-solving. *Minimization Reactions* (MR) refer to the degree to which parents minimize the seriousness of the situation or devalue the child’s problem or emotional response. In this study, all subscales presented good reliability with Cronbach alphas ranging between 0.72 and 0.91, except for DR (α = 0.50), which was excluded from the following analysis. As performed by Bost and colleagues [86], and since bivariate correlations revealed substantial associations between PR and MR (*r* = 0.68; *p* = 0.000) and between EE, EFR, and PFR (*r* = 0.48 to 0.66; all *p* = 0.000), composite scores of Positive and Negative Reactions were created to reflect total supportive emotion regulation (average of EE, EFR, and PFR subscales) and unsupportive emotion regulation strategies (average of PR and MR subscales). CCNES has been described as having good internal and test-retest reliability as well as good concurrent and construct validity [60].

#### 2.2.2. Children’s EF

Preschoolers’ EF was assessed using the Behavior Rating Inventory of Executive Function—Preschool Version (BRIEF-P) [76]. BRIEF-P is a rating scale used with caregivers (in our case mothers) to measure behavioral manifestations of preschool-aged children’s executive functions within everyday settings (home and preschool). It consists of 63 items, that evaluate 5 theoretical and empirical EF aspects: *Inhibit* assesses a child’s inhibitory control and impulsivity; *Shift* assesses the ability to move freely from one situation, activity, or aspect of a problem to another as the circumstances demand; *Emotional Control* measures the impact of EF difficulties on emotional expression; *Working Memory* measures the capacity to hold information for completing a task or generate goals and plans to achieve goals; and *Plan/Organize* measures the ability to manage current and future-oriented task demands within the situational context. The scales can form 3 indexes: *Inhibitory Self-Control* (ISCI), representing a child’s ability to modulate actions, responses, emotions, and behavior through appropriate inhibitory control; *Flexibility* (FI), representing a child’s ability to move flexibly among actions, responses, emotions, and behavior; and *Emergent Metacognition* (EMI), reflecting the child’s ability to maintain ideas and activities in working memory and to plan/organize problem-solving approaches. Caregivers are asked to answer each item regarding the behavior frequency in the last 6 months, using a 3-point Likert-type scale ranging from 1 = “Never” to 3 = “Always”. Higher values indicate lower levels of EF. All scales presented good reliability (Cronbach alphas ranging between 0.74 to 0.87 for mothers and between 0.69 to 0.89 for teachers) as did the indexes (Cronbach alphas between 0.84 and 0.90 for mothers and between 0.88 to 0.91 for teachers).

### 2.3. Procedure

This study is part of an ongoing research project “ChildObesity—Child obesity risk: the role of attachment, child’s temperament and self-regulation”, approved by the Ethics Committee of the ISPA—Instituto Universitário. The study was presented to the school boards to obtain the necessary authorizations for data collection. Informed consent was requested from mothers. After obtaining the mothers’ consent, teachers sent them the questionnaires to be completed at home. After completion, the mothers returned the questionnaires to teachers in a closed envelope, and teachers completed the BRIEF-P for the respective children.

### 2.4. Analytic Plan

Before our main analyses, descriptive statistics were explored using both mothers’ and teachers’ reports. Differences between boys and girls were tested using independent *t*-tests. Associations between mothers’ and teachers’ BRIEF-P reports, as well as between CCNES and BRIEF-P scales, were tested using Pearson’s correlation coefficient while controlling for child’s sex and age. Finally, a multiple regression was performed to explore negative emotion regulation contributions to child’s EF, where child’s sex, and age were included as covariables.

## 3. Results

### 3.1. Preliminary Analyses

Descriptive statistics are presented in Table 1. In general, mothers reported using more positive than negative reaction strategies. Both mothers and teachers described children as presenting good EF levels.

Significant sex differences were found for mothers’ BRIEF-P reports on Inhibit (*t*(134) = 3.28, *p* < 0.001; *M* = 1.62, *SD* = 0.29 for boys and *M* = 1.47, *SD* = 0.24 for girls), Shift (*t*(134) = 1.97, *p* < 0.05; *M* = 1.43, *SD* = 0.33 for boys and *M* = 1.33, *SD* = 0.24 for girls), and Emotional Control (*t*(134) = 2.92, *p* < 0.01; *M* = 1.55, *SD* = 0.31 for boys and *M* = 1.40, *SD* = 0.27 for girls) as well as for Inhibitory Self-Control (*t*(134) = 3.58, *p* < 0.001; *M* = 1.60, *SD* = 0.26 for boys and *M* = 1.45, *SD* = 0.22 for girls) and Flexibility index (*t*(134) = 2.77, *p* < 0.01; *M* = 1.49, SD = 0.28 for boys and *M* = 1.37, SD = 0.23 for girls); in all of them mothers described boys as presenting lower levels of EF. For teachers’ BRIEF-P reports, significant sex differences were also found but only for Inhibit (*t*(84) = 2.25, *p* < 0.05; *M* = 1.50, *SD* = 0.32 for boys and *M* = 1.35, *SD* = 0.29 for girls) and for Inhibitory Self-Control (*t*(84) = 2.00, *p* < 0.05; *M* = 1.46, *SD* = 0.31 for boys and *M* = 1.33, *SD* = 0.29 for girls). Teachers also described boys as showing lower levels of EF.

Results showed significant differences for firstborns, with mothers reporting the use of more Expressive Encouragement with them (*t*(121) = 3.06, *p* < 0.01; *M* = 5.57, *SD* = 1.11 for firstborns and *M* = 4.95, *SD* = 1.12 when not firstborns); no significant differences were found for either mothers’ or teachers’ BRIEF-P reports. There were significant differences when children had siblings, again only for CCNES, with mothers reporting using more Punitive Reactions (*t*(125) = 2.26, *p* < 0.05; *M* = 2.10, *SD* = 0.67 with siblings and *M* = 1.83, *SD* = 0.63 with no siblings) and Minimization Reactions when children had siblings (*t*(125) = 3.22, *p* < 0.01; *M* = 3.04, *SD* = 0.87 with siblings and *M* = 2.56, *SD* = 0.69 with no siblings). The same happened for the composite of Negative Reactions (*t*(125) = 3.09, *p* < 0.01; *M* = 2.57, *SD* = 0.70 with siblings and *M* = 2.19, *SD* = 0.59 with no siblings).

As children become older, mothers reported use of more Minimization Reactions (*r* = 0.21, *p* < 0.05; also reflected in the Negative Reactions composite, *r* = 0.19, *p* < 0.05), and attributed more Working Memory capacities to children (*r* = −0.27, *p* < 0.01; also reflected in the Emergent Metacognition index, *r* = −0.23, *p* < 0.01). Teachers described older children as having higher executive functions (Inhibit *r* = −0.27, *p* < 0.01, Shift *r* = −0.44, *p* < 0.001, Emotional Control *r* = −0.30, *p* < 0.01; as well as for the indexes Inhibitory Self-Control *r* = −0.32, *p* < 0.01 and Flexibility *r* = −0.41, *p* < 0.001).

Older mothers reported using more Negative Reactions (*r* = 0.24, *p* < 0.01), and this was true for both Punitive Reactions (*r* = 0.23, *p* < 0.01) and Minimization Reactions (*r* = 0.21, *p* < 0.05), but no significant correlations were found regarding BRIEF-P.

Finally, regarding the number of hours that the child spent in school, we found significant correlations with the mothers’ reports on using Positive Reactions (*r* = −0.29, *p* < 0.001) as well as for both Expressive Encouragement (*r* = −0.31, *p* < 0.001) and Problem-Focused Reactions (*r* = −0.24, *p* < 0.001), meaning that as children spend less time in school, more mothers report using positive strategies for dealing with children’s expression of distress. No other significant results were found for demographic variables.

In a smaller sample (*n* = 87), both mothers’ and teachers’ reports were used. Regarding BRIEF-P, as we can see in Table 2, mothers reported significantly higher scores for *Inhibit*, *Shift*, and *Emotion Control* scales (*t*(86) = 2.47, *p* < 0.05, *t*(86) = 4.10, *p* < 0.001 and *t*(86) = 2.64, *p* < 0.01, respectively) as well as for *Inhibitory Self-control* and *Flexibility* indexes (*t*(86) = 2.94, *p* < 0.01 and *t*(86) = 3.51, *p* < 0.001, respectively). There was a positive significant association between mothers’ and teachers’ reports on *Inhibit* and *Working Memory* scales (*r* = 0.36, *p* < 0.001 and *r* = 0.29, *p* < 0.01, respectively) as well as on *Inhibitory Self-control* and *Emergent Metacognition* indexes (*r* = 0.28, *p* < 0.01 and *r* = 0.28, *p* < 0.01, respectively).

### 3.2. Associations between CCNES and BRIEF-P

Correlations between mothers’ reactions to children’s distress and their reports on child EF were only significant for the *Negative Reactions* (including both *Punitive Reactions* and *Minimization Reactions*). Mothers reported using *Punitive Reactions* more as they described their children as having more EF problems (this was true for all BRIEF-P scales, as well as for the calculated indexes, *r* = 0.18 to 0.27). However, as we can see in Table 3, when controlling for the child’s sex and age, *Emotion Control* and *Working Memory* were no longer significant (*r* = 0.16, *p* > 0.05 and *r* = 0.17, *p* > 0.05, respectively). Regarding *Minimization Reactions*, we found a significant correlation with the *Inhibit* and *Emotion Control* scales as well as with the *Inhibitory Self-Control* and *Flexibility* indexes (*r* = 0.17 to 0.23). However, when we control for the child’s sex and age, only *Inhibit* and *Inhibitory Self-Control* remain significant (*r* = 0.23, *p* < 0.01 and *r* = 0.24, *p* < 0.01, respectively). Finally, as we can see in Table 3, *Negative Reactions* strategies were correlated with both the *Inhibit* and *Emotion Control* scales as well as with the *Inhibitory Self-Control* and *Flexibility* indexes, even when controlling for the child’s sex and age.

The associations between mothers’ reports on CCNES and teachers’ reports on BRIEF-P were also explored. As we can see in Table 4, there was a significant correlation for the *Negative Reactions* composite (including both *Punitive Reactions* and *Minimization Reactions*), even when controlling for the child’s sex and age. Additionally, for those children whose mothers report using more punitive strategies, teachers reported higher *Working Memory* problems (*r* = 0.31, *p* < 0.01). Teachers also reported lower *Working Memory* (*r* = −0.27, *p* < 0.05), *Shift* (*r* = −0.22, *p* < 0.05) and *Emergent Metacognition* (*r* = −0.22, *p* < 0.05) problems for those children whose mothers reported higher *Problem-Focused Reactions.*

### 3.3. Multiple Regressions with Mothers’ Negative Strategies

Regarding mothers’ reports, child’s sex and mother’s *Negative Reactions* (including both *Punitive Reactions* and *Minimization Reactions*) were significant predictors of the child’s *Inhibitory Self-Control* (*β* = 0.33; *p* < 0.01 and *β* = 0.30; *p* < 0.01, *R*^2^ = 0.19), *Flexibility* (*β* = 0.32; *p* < 0.01 and *β* = 0.28; *p* < 0.01, *R*^2^ = 0.17), and *Emergent Metacognition* (*β* = 0.25; *p* < 0.05, *R*^2^ = 0.14 and *β* = 0.26; *p* < 0.01, *R*^2^ = 0.16).

Regarding teachers’ reports, child’s age and mother’s *Negative Reactions* were significant predictors of children’s *Inhibitory Self-Control* (*β* = 0.18; *p* < 0.01 and *β* = 0.23; *p* < 0.05, *R*^2^ = 0.18). *Flexibility* was only significantly predicted by the child’s age (*β* = −0.40; *p* < 0.001, *R*^2^ = 0.19), whereas *Emergent Metacognition* was only significantly predicted by mothers’ *Negative Reactions* (*β* = 0.26; *p* < 0.05, *R*^2^ = 0.14).

## 4. Discussion

The main purpose of this study was to assess potential connections between caregivers’ reactions to children’s negative emotions and children’s EF. Partially consistent with our expectations, mothers’ use of negative emotion regulation responses to their children’s distress predicted children’s poor EF. When controlling for the child’s sex and age, we found the more mothers reported endorsing *Punitive Reactions* (i.e., use of verbal or physical punishment to control the child’s negative emotional expression), the more they described their children as having higher levels in almost all the BRIEF-P scales (except for the *Emotion Control* and *Working Memory* scales). When considering teachers’ reports on BRIEF-P, the results were similar, but with the addition that teachers also described children whose mothers reported higher use of punitive reactions as having more difficulties holding information in mind for completing a task or generating goals and plans to achieve goals (i.e., higher levels in the *Working Memory* scale). Furthermore, when controlling for the child’s sex and age, both mothers and teachers described children whose mothers reported higher use of *Minimization Reactions* (i.e., devaluation of the child’s negative emotional expression) as having more difficulties resisting impulses and inhibiting/modulating responses, emotions, and behavior (i.e., higher levels in the *Inhibit* scale and *Inhibitory Self-Control* index). In turn, mothers’ negative emotion regulation strategies—either punitive or minimization reactions—were also associated with more difficulties in impulse control, inhibition and modulation of responses, emotions, and behavior as well as the ability to move flexibly among those responses, emotions, and behavior (i.e., higher levels in the *Inhibit* and *Emotion Control* scales and in the *Inhibitory Self-Control* and *Flexibility* indexes). When considering teachers’ reports, mothers’ negative emotion regulation strategies were also associated with children’s poorer EF, namely higher levels in the *Inhibit*, *Working Memory*, and *Plan/Organize* scales, as well as the *Inhibitory Self-Control* and *Emergent Metacognition* indexes. Regression analyses indicated that mothers’ *Negative Reactions* accounted for a significant proportion of the variance in *Inhibitory Self-Control* (for both mothers and teachers), *Flexibility* (only for mothers), and *Emergent Metacognition* (for both mothers and teachers) indexes. Although this seems to be the first study to assess links between parental emotional responsiveness and children’s EF, these findings are consistent with previous studies supporting the role of caregivers in children’s EF (e.g., [53,54,82,87,88,89,90,91,92]). For example, Bernier and colleagues [53] proposed that parental responsiveness could promote higher levels of children’s EF by affecting children’s neurobiological structure development and providing a social environment for the child to observe and practice positive regulatory strategies related to EF. In this sense, as parental responsiveness to children’s emotions contributes to children’s development of emotion regulation (e.g., [1,62,66,67]), and, in turn, emotion regulation influences EF [44], we believe that caregivers’ responses to children’s emotions could have an important role in children’s EF. Therefore, it is possible that when caregivers use non-supportive emotional responses to deal with children’s negative emotions, they provide an inappropriate social environment for children to practice and develop their emotion regulation skills, which, in turn, could negatively affect their EF.

Contrary to what was expected, mothers’ use of positive emotion regulation responses was not linked to children’s EF. Only when considering teachers’ reports we found that mothers’ higher use of *Problem-Focused Reactions* (i.e., helping the child to solve the problem) was associated with children’s higher ability to shift from one situation/activity to another or solve problems flexibly as well as to hold information in mind for completing a task (i.e., lower levels in the *Shift* and *Working Memory* scales). Thus, this reflected children’s higher ability to initiate, plan, organize, implement, and maintain future-oriented problem solving (i.e., lower levels in the *Emergent Metacognition* index). A possible explanation for why only negative emotional responsiveness was related to children’s EF is that negative interactions may be more impactful than positive ones [93,94]. For example, Zemp and colleagues [94] suggest that parental positive interactions should be expressed at least twice as much as negative interactions to protect children from adjustment problems. Their results showed that when negativity outweighed positivity, children scored significantly higher in externalizing problems. In this sense, it is important to maintain a high ratio between caregivers’ supportive versus unsupportive responses to children’s distress to prevent the adverse effects of negative emotional responsiveness on children’s EF. Future studies are needed to assess the unique impact of caregivers’ positive and negative emotional responses on children’s executive functions.

Regarding sex differences in EF, both mothers and teachers described boys as presenting poorer EF. Mothers and teachers reported higher levels on the *Inhibit* scale and the *Inhibitory Self-Control* index, which could mean that they perceive boys as having more problems with impulse control and inhibition of responses, emotions, and behavior than girls. Mothers also reported higher levels on the *Shift* and *Emotional Control* scales and on the *Flexibility index*, which indicates they perceive boys as having more difficulties in transitions between situations/activities, controlling emotional responses, and moving flexibly among responses, emotions, and behavior. These findings corroborate previous studies in other cultures [83,95,96,97,98,99]. However, they are inconsistent with results from other Portuguese samples that found no significant sex differences [100]. This inconsistency could be due to the use of different EF measures (i.e., performance-based measures and rating measures).

We also found differences in mothers’ reactions to children’s negative emotions concerning children with and without siblings. Mothers reported higher use of negative emotion regulation strategies for children with siblings. This finding is consistent with previous research showing that children with no siblings receive more positive responses from their caregivers. Additionally, caregivers of children with no siblings tend to be more responsive to their children than caregivers with more than one child [101]. A possible explanation for this relies on the theory of resource dilution, which suggests that with the addition of each child, the family resources decline [102,103,104]. Consequently, the only child receives, for example, more attention, concern, care, support, and interaction from caregivers, which, in turn, could result in later better socio-emotional competence and emotion regulation skills [105,106].

Some limitations bear noting. First, this study used cross-sectional and concurrent data, not allowing for inferences about the causality or directionality of the relations assessed. Future longitudinal studies are needed to disentangle the relation between caregivers’ responses to children’s emotions and children’s EF over time, considering the possibility of bidirectional relationships and age effects [65]. Second, data are all self-report, and so may be susceptible to reporting bias [107,108]. Additionally, mothers may underestimate their use of particular responses to children’s emotions or be unaware of how often they use certain responses [109]. In this sense, future research would benefit from a multimethod approach that includes observational measures to obtain more accurate information about the emotion regulation strategies used by caregivers. Third, like most studies about parental reactions to children’s emotions, we only considered mothers’ responses to children’s negative emotions. However, responses to children’s positive emotions also have important implications for their development [110], so future research should consider parental responsiveness to children’s positive emotions. Lastly, as previously referred, other variables might play a role in the relationship between caregivers’ emotional responses and children’s EF, namely children’s emotion regulation and its multidimensional nature. Thus, future studies should consider the mediation role of emotion regulation. Despite these limitations, findings from this study are novel and have important implications for future research. Our data offer preliminary support for emerging research that takes into account the relationship between parental emotional responsiveness and children’s EF. Another strength of our study is that ratings from teachers were included. As previously mentioned, teachers may be more reliable reporters of children’s EF given the higher demand for children’s executive functions at school [81]. In addition, teachers may have a better knowledge of what behaviors are normative as they are more familiar with the type of behaviors expected at specific periods of development and have a group of other children for comparison [111]. Our data also have implications for practice. The preschool years are a time of rapid growth and development of executive functions, and it is important to assess difficulties during this period given the significant influence of early EF across development, including adulthood [6]. In this sense, the preschool period provides an opportunity for prevention and intervention programs targeting children’s EF, and findings from this study suggest that such programs could be enhanced by assessing parental emotional responsiveness.

## Figures and Tables

**Table 1:** Means and standard deviations regarding CCNES and BRIEF-P.

		Mother (*n* = 136)		Teacher (*n* = 87)	
		*M*	*SD*	*M*	*SD*
CCNES	Distress Reactions	2.74	0.60		
	Punitive Reactions	2.01	0.68		
	Minimization Reactions	2.89	0.84		
	Expressive Encouragement	5.26	1.12		
	Emotion-Focused Reactions	5.97	0.69		
	Problem-Focused Reactions	5.98	0.72		
	Negative Reactions	2.44	0.70		
	Positive Reactions	5.74	0.72		
BRIEF-P	Inhibit	1.56	0.28	1.44	0.32
	Shift	1.39	0.29	1.23	0.26
	Emotional Control	1.48	0.30	1.36	0.38
	Working Memory	1.41	0.30	1.40	0.33
	Plan/Organize	1.43	0.29	1.46	0.33
	Inhibitory Self-Control	1.53	0.25	1.40	0.30
	Flexibility	1.43	0.26	1.29	0.29
	Emergent Metacognition	1.42	0.28	1.42	0.31

*M* = mean; *SD* = standard deviation; CCNES = Coping with Children’s Negative Emotions Scale; and BRIEF-P = Behavior Rating Inventory of Executive Function—Preschool Version.

**Table 2:** Means and standard deviations for mothers’ and teachers’ reports on BRIEF-P.

	Mother		Teacher				
	*M*	*SD*	*M*	*SD*	Mean Difference	*t*	*p*
Inhibit	1.53	0.28	1.43	0.32	0.09	2.47	<0.05
Shift	1.39	0.29	1.23	0.26	0.16	4.10	<0.001
Emotional Control	1.49	0.30	1.36	0.38	0.14	2.64	<0.01
Working Memory	1.37	0.28	1.40	0.33		n.s.	
Plan/Organize	1.39	0.28	1.46	0.33		n.s.	
Inhibitory Self-Control	1.51	0.26	1.40	0.30	0.11	2.94	<0.01
Flexibility	1.44	0.26	1.29	0.29	0.15	3.51	<0.001
Emergent Metacognition	1.38	0.26	1.42	0.31		n.s.	

*M* = mean; *SD* = standard deviation; n.s. = no significant differences.

**Table 3:** Associations between mother’s report on CCNES and BRIEF-P scales, controlling for child’s sex and age.

			BRIEF-P						
		Inhibit	Shift	Emotional Control	Working Memory	Plan/Organize	Inhibitory Self-Control	Flexibility	Emergent Metacognition
	PN	0.30 ***	0.18 *	0.16	0.17	0.19 *	0.28 **	0.19 *	0.19 *
CNNES	MR	0.23 **	0.09	0.17	0.12	0.12	0.24 **	0.15	0.13
	EE	−0.07	−0.04	0.00	−0.08	−0.04	−0.05	−0.02	−0.07
	EFR	−0.06	−0.03	−0.11	−0.08	−0.04	−0.09	−0.08	−0.07
	PFR	−0.02	−0.08	−0.07	−0.08	−0.07	−0.05	−0.09	−0.08
	NR	0.29 **	0.14	0.18 *	0.16	0.17	0.28 **	0.19 *	0.17
	PR	−0.06	−0.06	−0.06	−0.09	−0.06	−0.07	−0.07	−0.08

* *p* < 0.05; ** *p* < 0.01; *** *p* < 0.001; CNNES: PN = Punitive Reactions, MR = Minimization Reactions, EE = Expressive Encouragement, EFR = Emotion-Focused Reactions, PFR = Problem-Focused Reactions, NR = Negative Reactions Composite, PR = Positive Reactions Composite.

**Table 4:** Associations between mothers’ reports on CCNES and teachers’ reports on BRIEF-P scales, controlling for child’s sex and age.

			BRIEF-P						
		Inhibit	Shift	Emotional Control	Working Memory	Plan/Organize	Inhibitory Self-Control	Flexibility	Emergent Metacognition
	PN	0.23 *	0.23 *	0.18	0.31 **	0.25 *	0.24 *	0.23 *	0.30 **
CNNES	MR	0.28 **	0.07	0.10	0.16	0.14	0.23 *	0.10	0.16
	EE	−0.11	−0.02	−0.06	−0.14	−0.11	−0.10	−0.05	−0.14
	EFR	−0.21	−0.02	−0.05	−0.05	−0.04	−0.16	−0.03	−0.01
	PFR	−0.20	−0.22 *	−0.12	−0.27 *	−0.11	−0.18	−0.16	−0.22 *
	NR	0.28 **	0.17	0.13	0.28 **	0.23 *	0.25 *	0.17	0.27 *
	PR	−0.20	−0.09	−0.09	−0.18	−0.08	−0.17	−0.09	−0.15

* *p* < 0.05; ** *p* < 0.01; CNNES: PN = Punitive Reactions, MR = Minimization Reactions, EE = Expressive Encouragement, EFR = Emotion-Focused Reactions, PFR = Problem-Focused Reactions, NR = Negative Reactions Composite, PR = Positive Reactions Composite.

## Data Availability

The raw data supporting the conclusions of this article will be made available by the authors, without undue reservation.
